# 

**Published:** 1908-04-04

**Authors:** 


### BOOKS RECEIVED.

Methuen and Co.
"Diseases of Occupation." By T. Oliver, M.D.

T. Nelson and Sons.
"The Octopus." By F. Norris. Nelson's Library.

Rebman, Ltd.
"Morag, the Seal." By J. W. Brodie Innes.
"A Daughter of Belial." By Basil Tozer.
"The Wife." By Horace W. C. Newte.
"The Irony of Marriage." By Basil Tozer.

The Scientific Press, Ltd.
"Burdett's Hospitals and Charities, 1908."

### Tuberculosis and the Milk Supply.

A conference was recently held at the Town Hall, Manchester, of delegates representing the Public Health Committees of the cities of Liverpool, Manchester, Birmingham, Leeds and Sheffield in regard to the danger to man of milk containing the living infection of tuberculosis.

The Conference passed the following resolution:—

"That having regard to the experience of the five towns whose delegates have conferred on this subject, and also to the return recently made to the House of Commons at the request of Dr. Rutherford, a representation be made to the Presidents of the Local Government Board and the Board of Agriculture and Fisheries with a view to inducing these Boards to take effective steps to enforce uniformly throughout the country proper and suitable inspection of dairies and cowsheds, and for regulating the construction of such dairies and cowsheds so as to ensure cleanliness and suitable hygienic conditions; and, further, that the Government be respectfully asked to include in their prospective legislation dealing with milk clauses calculated to bring about the eradication of tuberculosis from bovines within a measurable period of years."

It is intended to present this resolution to both the Local Government Board and the Board of Agriculture.

### The Medical Schools of London.

Under the title of "London's Medical Schools," the *Graphic* has begun a series of illustrated articles, which should prove of much interest to the medical profession. The issue for March 28 contains the first instalment, which deals with the medical school of St. Bartholomew's Hospital. The illustrations, thirteen in number, occupy a complete double page, and are admirably reproduced from recent photographs. They convey a very good idea of the various departments of the medical school, and of the excellent accommodation for students in the new buildings at St. Bartholomew's. The leterpress gives a brief outline of the course of study pursued at the medical school, and of the arrangements of the Students' Union.

We look forward with pleasure to the succeeding numbers of this series.

### THE GRADUATES' MEDICAL SCHOOLS.

DIARY FOR WEEK APRIL 4 TO 11.

### MEDICAL GRADUATES' COLLEGE AND POLYCLINIC, Chenies Street, W.C.

**At 5.15 p.m.**

**April 6,** Dr. H. Walsham, **The X-Rays in the Diagnosis of Diseases of the Chest.**

**April 7 and 8,** Dr. C. O. Hawthorne, **The Clinical Anatomy and Physiology of the Nervous System.**

**April 9,** Dr. Oliver (Harrogate), **Recent Studies in Arteriosclerosis.**

THE HOSPITAL. *April* 4, 1908.

